# Recreational gymnastics exercise of moderate intensity enhances executive function in Chinese preschoolers: A randomized controlled trial

**DOI:** 10.1002/pchj.786

**Published:** 2024-06-23

**Authors:** Jianhua Zhang, Jiacheng Lu, Youbin Sun, Ji Li

**Affiliations:** ^1^ Academic Division of Humanities and Social Sciences Beijing Sport University Beijing China; ^2^ College of Physical Education Shanxi University Taiyuan China; ^3^ English Teaching Office Shangqiu Vocational Education Centre Shangqiu China; ^4^ Academic Division of Olympic Sports Beijing Sport University Beijing China

**Keywords:** executive function, moderate intensity, physical activity, preschooler, recreational gymnastics

## Abstract

The current study aimed to investigate the impact of recreational gymnastics on executive function in Chinese preschoolers, with a focus on gymnastics potential to enhance core components of executive function. A total of 63 preschool children who received full‐time education were randomly assigned to either an intervention group (*N* = 31, mean age = 66.27 months, *SD* = 3.12 months) or a control group (*N* = 32, mean age = 66.79 months, *SD* = 3.34 months). The intervention group engaged in recreational gymnastics for 60 min, three times a week for 12 weeks. Meanwhile, the control group continued with their typical outdoor activities at kindergarten and did not participate in any organized sports. The intervention program was primarily conducted through group play and was facilitated by teachers who underwent standardized training. Various simple and complex tasks were utilized to evaluate delay gratification (Snack delay and Wrapped gift), inhibitory control (Stop signal task and Circle drawing task), working memory (Letter memory task and Keep track task), and cognitive flexibility (Go/No‐Go task and Dots task). The analysis of covariance revealed that the children who participated in the intervention outperformed the control group on most simple and complex executive function tasks. Specifically, these children demonstrated an enhanced ability to regulate persistent responses, process and update information, and manage high cognitive conflict. The findings of this investigation lend support to the hypothesis that moderate‐intensity recreational gymnastics is an efficacious means of enhancing executive function in early childhood. Future research should employ a larger sample size, incorporate a long‐term follow‐up design, and utilize a multi‐method approach to further substantiate the impact of moderate‐intensity gymnastics on the executive function of young children, as well as to investigate its underlying mechanism and generalizability.

## INTRODUCTION

Executive function (EF) is a comprehensive term encompassing a range of sophisticated cognitive processes, including inhibitory control, working memory, and cognitive flexibility. These processes regulate and govern cognition in diverse task environments that are constantly changing (Traverso et al., 2015). EF enables individuals to make optimal decisions in the face of complex and varied life demands (Miyake et al., 2000). In addition to the conventional cognitive model, the emotional facet of EF is also conceptualized. Zelazo and Müller (2011) distinguish between relatively “hot” emotional development and more pure “cool” cognitive development in EF. According to their research, hot EF is linked with processes that are triggered by emotional conditions, such as delayed gratification and emotional decision‐making. In contrast, cool EF involves cognitive skills that operate in neutral situations, such as working memory. Zelazo and Carlson (2020) proposed that hot and cold EFs typically work in tandem as part of a broader adaptive system.

EF serves as the fundamental pillar of cognitive and behavioral development in preschool‐aged children (Turnbull et al., 2013). First, self‐control constitutes the central element of EF, encompassing the capacity to regulate impulses, delay gratification, and exhibit patience, among other skills (van Ginneken, 2017). These proficiencies are indispensable for fostering social interaction and learning in preschoolers. By means of exercising self‐control, preschoolers can actively engage in collaborative activities, patiently await their turn, and adhere to established rules, thereby cultivating positive social skills and behavior. Second, EF plays a crucial role in learning and school readiness (Blair, 2002). Working memory, the ability to temporarily store and process information, is indispensable for the acquisition of academic skills such as reading, mathematics, and problem‐solving (Gathercole et al., 2006). The developmental level of working memory in preschool children is closely associated with their future academic accomplishments (Bull et al., 2008). Additionally, EF contributes to the cultivation of cognitive flexibility, which refers to children's capacity to adapt to setbacks and challenges (Anderson, 2002). Preschoolers often encounter novel situations, such as acquiring new concepts, adjusting to unfamiliar environments, and coping with emotional difficulties (Kelly & Emery, 2003). With well‐developed EF abilities, children are more inclined to actively respond to these challenges while fostering positive emotions and adaptive behaviors (Nesbitt et al., 2015). Finally, EF also encompasses decision‐making skills, which are vital for daily life choices and problem‐solving tasks (Del Missier et al., 2012). Preschoolers frequently face decisions ranging from selecting playmates to resolving conflicts (Green & Rechis, 2006). Enhanced executive capabilities enable them to evaluate options effectively, anticipate consequences accurately, and make informed decisions (Helfat & Peteraf, 2015). In recent decades, researchers have dedicated their efforts to investigating strategies for enhancing and fostering the EF of preschool children, with the aim of positively influencing their future learning outcomes and behavioral development.

In this domain, research has yielded valuable insights into the impact of various intervention methods on the EF of preschool children, encompassing cognitive training (Wass, 2015), gamification interventions (Liu et al., 2022), and sports activities (McNeill et al., 2020). However, it is noteworthy that there exists a paucity of studies investigating the effects of gymnastics on the EF of preschool children, particularly within the framework of randomized controlled trials (RCTs). RCTs are considered one of the most rigorous research designs as they enable researchers to accurately evaluate the influence of gymnastics on preschoolers' EF by randomly assigning participants to either an intervention or a control group. This approach helps mitigate confounding factors and ensures reliable and scientifically sound research findings.

Recreational gymnastics is regarded as a coordinated physical activity owing to its emphasis on precise coordination and balance among different body parts (Busquets et al., 2021). First, recreational gymnastics necessitates the execution of a diverse range of intricate and highly coordinated movements, including tumbling, balancing, and rotating. These movements demand impeccable timing, strength control, and the harmonious integration of all bodily components for successful execution. Second, gymnastics underscores spatial perception and awareness within the body. Participants must possess a clear understanding of their body's position and orientation during movement in order to execute complex maneuvers within confined spaces. This necessitates a high degree of coordination and precision. Additionally, recreational gymnastics places great importance on equilibrium and stability. Participants are required to perform actions on various apparatuses such as the balance beam while maintaining optimal balance through effective control over bodily coordination to prevent falls or loss of equilibrium. Lastly, recreational gymnastics mandates the completion of intricate sequences within limited timeframes, which calls for rapid cognitive processing and motor response to ensure seamless coordination and consistency in movement. Recreational gymnastics offers numerous advantages, encompassing the promotion of physical health (Kiuchukov et al., 2019), coordination (Hiley et al., 2019), flexibility (Niaradi et al., 2019), and quality of life (Wahyuni & Arifiati, 2021). Consequently, it not only holds potential for positively impacting EF but also contributes to the holistic development of preschool children. Nevertheless, further research is imperative to comprehend the prospective short‐ or long‐term effects of this intervention on EF in young children.

Previous research has demonstrated that engaging in moderate physical activity can enhance children's cognitive function (Tandon et al., 2016). However, determining the optimal exercise intensity remains a topic of debate. High‐intensity exercise may exceed young children's physical capacity, leading to potential physiological and psychological risks, while low‐intensity exercise may not elicit sufficient physiological responses to achieve the desired training effects. Therefore, we have chosen a medium intensity level for children participating in recreational gymnastics. Moreover, medium‐intensity physical activity is inherently captivating and demanding (Piao et al., 2022), thus capturing children's attention and fostering their active involvement, ultimately enhancing the maneuverability of the study and bolstering the reliability of its findings.

Therefore, the primary objective of this study is to compare the disparities in EF between preschool children engaged in a 12‐week moderate‐intensity recreational gymnastics intervention and those in the control group. Based on the study's objective, we hypothesize that participation in medium‐intensity recreational gymnastics has a significant impact on the EF of preschool children, specifically resulting in significantly higher EF scores in the recreational gymnastics group compared with the control group.

## MATERIALS AND METHODS

### Participants and design

**FIGURE 1:**
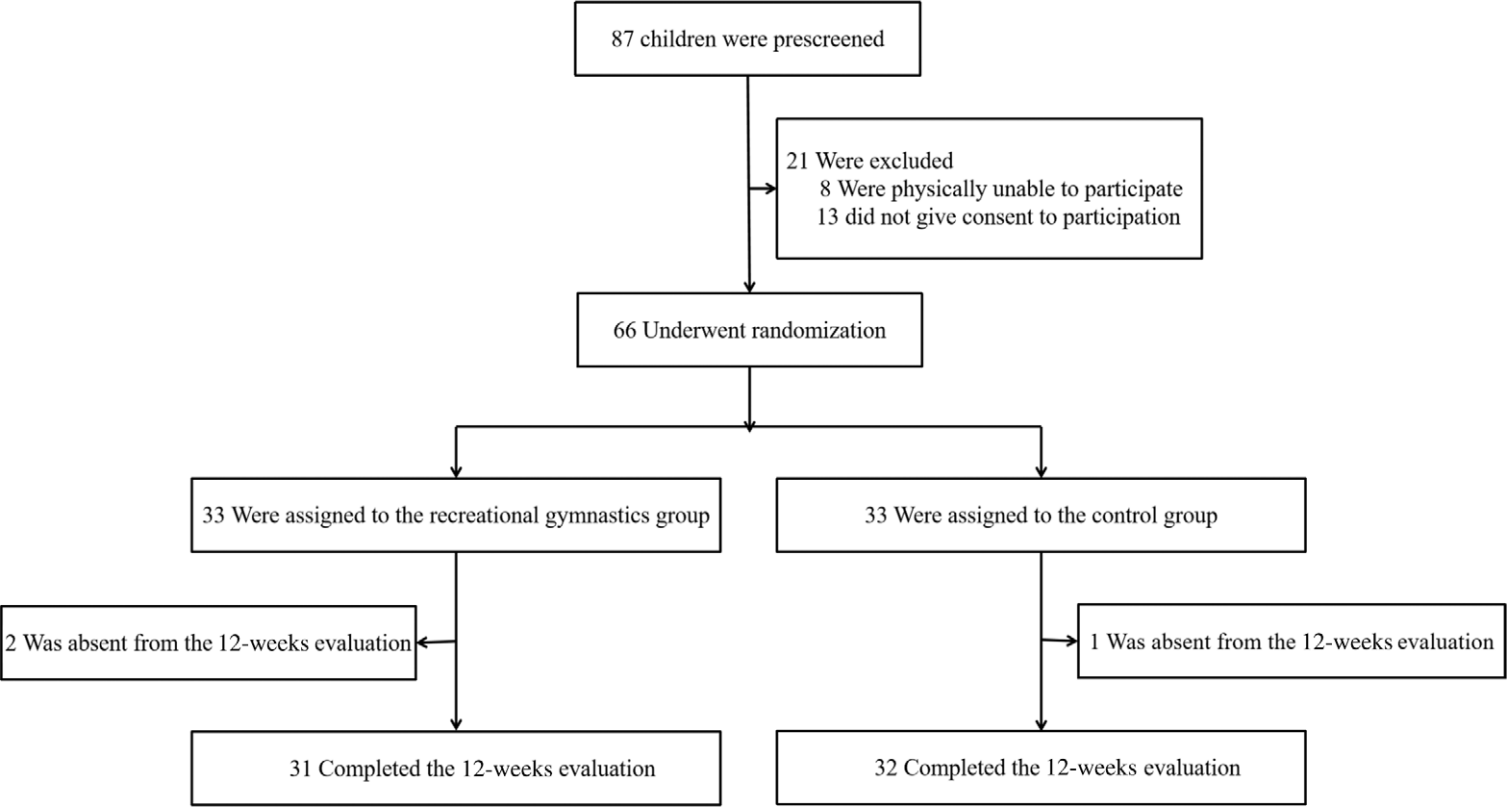
Screening, randomization, and completion of 12‐week evaluations.

As depicted in Figure 1, a total of 87 preschoolers from three classes were initially screened for participation. The participants in this study are preschool children aged 5–6 years, representing the final year of kindergarten. However, 13 parents did not agree to their children's involvement, and an additional eight children were excluded owing to physical discomfort. The remaining 66 children received parental consent, and socio‐demographic information was obtained along with behavioral data through the completion of two brief questionnaires. Subsequently, a random selection process was employed to assign half of the participants (where there were 20, 24, and 22 individuals in the three classes) to the intervention group (IG), while the remaining individuals were allocated to the control group (CG). The implementation of the process aims to ensure a consistent classroom environment for both IG and CG participants, thereby minimizing discrepancies in experimental outcomes. The researchers were not involved in the allocation of participants to either the intervention group or the control group. Following randomization, each group consisted of 33 children. The analysis using the *χ* ^2^ test and *t* test revealed that there were no significant differences between the two groups in terms of age, sex, body mass index (BMI), intelligence, socioeconomic status (SES), and so on. The demographic data of the children are shown in Table 1.

**TABLE 1:** Comparison of socio‐demographic characteristics and other variables between the IG and CG at baseline level, M (*SD*).

Variables	IG	CG	Difference	
Age (months)	66.27 (3.12)	66.79 (3.34)	*t*_(1,61)_ = −0.135, *p* = .851	ND
Sex distribution of female (%)	48.39 (2.14)	54.84 (2.78)	*X* ^2^_(1,61)_ = 0.726, *p* = .420	ND
BMI	15.60 (1.76)	16.42 (1.83)	*t*_(1,61)_ = −0.335, *p* = .728	ND
Raven matrices	15.91 (2.73)	16.28 (3.16)	*t*_(1,61)_ = −0.892, *p* = .552	ND
Socio‐economic status	6.24 (1.51)	6.37 (1.78)	*t*_(1,61)_ = −0.435, *p* = .212	ND
Dis‐attentive behaviors	6.89 (1.07)	7.03 (0.92)	*t*_(1,61)_ = −0.175, *p* = .933	ND
Hyperactive‐impulsivity symptoms	6.45 (1.32)	6.21 (1.25)	*t*_(1,61)_ = 0.215, *p* = .945	ND

Abbreviations: BMI = body mass index, calculated as weight (kg)/height (m)^2^; CG = control group; IG = intervention group; M (*SD*) = mean (standard deviation); ND = no difference.

Participants underwent a 2‐day testing protocol at baseline and post‐intervention, during which they were instructed to abstain from caffeine and exercise on the day of testing. The assessment of EF was conducted by a trained researcher who was blinded to group allocation, while the primary investigators remained uninvolved in measurement procedures. In order to ensure data accuracy and analysis reliability, we established exclusion criteria: students who were absent for a duration exceeding 2 weeks, equivalent to more than six classes, would be excluded from the final data analysis. Three children (one in the control groups and two in the intervention group) were excluded from analysis owing to prolonged absences exceeding 2 weeks. The final sample comprised 63 children, with 31 in the intervention group and 32 in the control group.

This study was conducted in accordance with the ethical guidelines of the Declaration of Helsinki and was approved by the Ethics Committee for Exercise Science Experiments at Beijing Sport University (Agreement code 2022015H). The inclusion criteria stipulated that participants must possess normal or corrected‐to‐normal vision, exhibit no color blindness, and demonstrate right‐handed dominance.

### Intervention program

The intervention program aimed to enhance EF skills through a series of recreational gymnastics activities. The content of the recreational gymnastics in this study primarily focused on providing enjoyable and engaging methods for sports participation, rather than on emphasizing skill development. The study employed a double‐blind design to minimize potential subjective biases among experimental participants. Specifically, during the intervention, the majority of activities were conducted in pairs or small groups. The comprehensive intervention consisted of various moderate‐intensity recreational gymnastics activities that took place three times a week for 12 weeks. Each session lasted approximately 60 min and was held on Monday, Wednesday, and Friday from 08:55 to 09:55 in the kindergarten playground. Before engaging in recreational gymnastics, the instructor provided a demonstration and explanation of the activity, followed by an introduction to safety considerations. During practice sessions, students were supervised by well‐trained instructors who offered necessary guidance. According to the exercise guidelines provided by the American College of Sports Medicine, moderate intensity is typically defined as ranging from 60% to 70% of an individual's maximum heart rate. (American College of Sports Medicine, 2012). Each participant's maximum heart rate was estimated by subtracting their age from 220, as described in Gibson et al. (2019). A Polar FT2 HR monitor was utilized to track heart rate during exercise, with an alarm sounding if the heart rate exceeded the designated range. Exercise intensity was adjusted through changes in speed or load while ensuring that the heart rate remained within the specified range. The rest periods during exercise were flexibly scheduled based on the participants' heart rate changes. Owing to resource constraints, a random selection of only five children underwent heart rate monitoring prior to the commencement of each class. Meanwhile, children in the control group remained indoors within the classroom and were exposed to designated educational cartoons during the same timeframe. The daily activities of both groups remained consistent. Each recreational gymnastics session consisted of the following components.

1. The warm‐up segment primarily consisted of group play activities, such as animal mimicry for locomotion and jumping, lasting a total of 10 min.
2. The exercise component primarily comprised gross motor movements that integrate balance, agility, coordination, speed, and explosive power control. These include forward and backward rolls on a mat, multiple forms of jumping on a mini trampoline, as well as various forms of walking on the balance beam, for a total duration of 45 min.
3. The relaxation segment primarily involved comprehensive body stretching, encompassing both upper and lower limb muscle groups, with a duration of 5 min in total.

### Measurements

The time interval between our post‐test and the last training session was set at 24 h to mitigate any residual acute effects from the previous exercise regimen. The tests were conducted individually in a quiet environment devoid of distractions, with multiple tasks per EF being selected based on the following criteria: (1) previous usage or reliance on such tasks in research; (2) minimal impact from non‐executive abilities; (3) differentiation from intervention activities; and (4) ranging from simple motion control to conditions with high cognitive conflict.

#### Hot EF tasks

##### Snack delay task

This task is commonly employed to evaluate a child's capacity for delayed gratification (Kochanska et al., 2000). The child, with hands on a mat on the table, waited for the experimenter to ring the bell before retrieving a candy from under a transparent cup (four trials, with delays of 10, 20, 30 and 10 s). In mid‐delay, the experimenter lifted the bell but did not ring it. Coding reflected the ability to delay (1 = pre‐bell candy consumption, 2 = post‐bell candy consumption, 3 = pre‐bell candy contact, 4 = post‐bell candy contact, 5 = pre‐ringing bell or cup contact, 6 = post‐ringing bell or cup contact or cup and bell simultaneous contact, and finally, 7 = waiting for the ringing of the bell). The higher the score, the greater the children's capacity for delayed gratification.

##### Wrapped gift task

This task is designed to evaluate a child's capacity for delaying gratification and inhibiting undesirable behavior (Kochanska et al., 1996). The examiner first inquires whether the child desires a gift. Following the child's response, the examiner states, “I can provide you with the gift, but I must wrap it first. You are not permitted to peek until it has been wrapped.” Subsequently, the child is instructed to turn away from the experimenter while they noisily wrap the present within 60 s. In the course of the experiment, the researcher monitored whether or not the child engaged in peeking behavior. Coding reflected the ability to delay (1 = child refrains from turning back, 2 = child turns back but then returns gaze forward, 3 = child looks over shoulder to view stimulus, 4 = child turns head partially but less than 90°, 5 = child does not attempt to peek). The time elapsed before the initial peek and total score within a 1‐min interval were both documented. The greater the delay time and the higher the score, the stronger the child's ability to delay gratification.

#### Inhibitory control

##### Stop signal task

This task is commonly employed to evaluate a child's capacity for inhibiting irrelevant interference stimuli (Livesey et al., 2006). The task was presented using E‐Prime 2.0 software. The distance between the child and the screen measured approximately 37 cm. When presented with left or right arrows on the computer screen, children were required to press the corresponding keys. However, upon hearing a “beep” as a stop signal, they were instructed to withhold their response and refrain from pressing any keys. Every time two arrows simultaneously appeared, the consistent condition was when they pointed in the same direction, while the inconsistent condition was when they pointed in opposite directions; a “beep” served as the stop signal. Each of these three conditions had 16 items, and after two practice rounds for each condition, a formal test consisting of 48 randomly presented items commenced. The experiment commenced with a “+” symbol displayed at the center of the screen for a duration of 500 ms, followed by the presentation of experimental stimuli at intervals of 1500 ms. In cases where no response was recorded within 2000 ms, the subsequent stimulus was automatically presented. The participant's average reaction time and the total number of correct responses to all stimuli were recorded.

##### Circle drawing task

This task is utilized to assess the motor inhibition of children's continuous responses (Marzocchi et al., 2008). A circular shape with a diameter of 17 cm was presented on paper, and the child was required to trace it from start to finish using a pencil. The task was performed twice, first with a neutral instruction to “draw a circle”, and then with an inhibitory instruction to “draw again, but at a slower pace”. The greater the temporal discrepancy, the stronger the inhibitory capacity of children towards continuous response. The duration of each test (in seconds) was recorded, and the score was calculated as the deceleration relative to the total time. The formula used for calculation was (T2−T1)/(T2 + T1), where T1 and T2 represent the recorded times of the first and second tests, respectively.

#### Working memory

##### Letter memory task

This task effectively assesses the updating function of children's working memory (Kusak et al., 2000). The task was presented using E‐Prime 2.0 software (Psychology Software Tools, Pittsburgh, Pennsylvania, USA). The distance between the child and the screen measured approximately 37 cm. Children must visually perceive a sequence of five letters and accurately articulate them according to prescribed rules. For example, when presented with the letters L, R, Z, M, and Q in sequence at the center of the screen, a child will accurately report them in order: L, LR, LRZ, RZM, ZMQ. The letters were presented at a rate of one per second, and each child was awarded one point for accurately reproducing the required letter string. After two practice sessions, eight formal tests were conducted, with a maximum score of eight points.

##### Keep track task

This task aims to assess a child's capacity for information updating (St Clair‐Thompson & Gathercole, 2006). The task was presented using E‐Prime 2.0 software. The distance between the child and the screen measured approximately 37 cm. The child was presented with a series of pictures, each belonging to one of five categories: colors (yellow, red, blue, black), animals (dog, cat, panda, rabbit), vehicles (train, bicycle, plane, car), clothing items (socks, skirt, trousers, shoes) and fruits (strawberry, grapes, banana, apple). The stimuli were presented in octets at the center of the computer display, with each belonging to distinct category, such as colors, animals, vehicles, clothing and fruits. At the bottom of the screen, three categories were consistently displayed, and it was incumbent upon the child to keep track of the most recent category among them. The center of the computer screen contained various irritants, such as yellow, pandas, socks, black, grapes, shoes, rabbits and apples. The categories displayed at the bottom of the screen included colors, animals, and fruit. Subsequently, the child was tasked with recalling the final items for each category, which were correctly identified as black for colors, rabbit for animals, and apple for fruit. The stimuli were presented sequentially at a rate of one per second. Following the appearance of the last stimulus, three categories were displayed consecutively at 1‐s intervals. Subsequently, there was a 3000‐ms interval during which children were required to provide their responses. One point was awarded to children who provided an exact response that met requirements. Following two practice sessions, nine formal assessments were administered, with a maximum score of nine points. The stimulus was presented centrally on the computer screen.

#### Cognitive flexibility

##### Go/No‐Go task

This task, which was adapted from Zelazo's work (Zelazo, 2006), is a widely employed method for assessing children's cognitive flexibility. The task was presented using E‐Prime 2.0 software. The distance between the child and the screen measured approximately 37 cm. The child was required to press the space‐bar in three specific situations: (1) press the space‐bar when a red figure appears and refrain from doing so when a blue figure appears (30 items: 12 red rabbits, 12 red cars, 3 blue rabbits, and 3 blue cars); (2) press the space‐bar when there is a rabbit, and refrain from doing so when there is a car (30 items: 12 red rabbits, 12 blue cars, 3 red rabbits, and 3 blue cars); (3) press the space‐bar when there is a red rabbit, and refrain from doing so when other images are displayed (40 items: 32 red rabbits, 4 red cars, 2 blue rabbits, 2 blue cars). Under all three experimental conditions, there was a consistent Go response rate of 80%. The duration of stimulation was fixed at 3000 ms, followed by a blank screen lasting for 1000 ms. The total number of correct responses in the No‐Go condition and the mean response time across all three conditions were computed.

##### Dots task

This task, adapted from Davidson et al. (2006), requires children to flexibly switch between different rules based on the presented stimuli (Diamond & Lee, 2011). The task was presented using E‐Prime 2.0 software. The distance between the child and the screen measured approximately 37 cm. Specifically, a sun or moon stimulus appears on either side of the screen, and the child needs to press either the same‐side button for suns or the opposite‐side button for moons. Each of these two conditions had 10 items, and after two practice rounds for each condition, a formal test consisting of 20 randomly presented items commenced. A 500‐ms warning cross was presented in the center of the blank screen, followed by a 500‐ms stimulus, and then an empty‐screen period lasting for 3000 ms. The number of accurate responses and average response latency for each child were documented.

##### Fluid intelligence

The Colored Progressive Matrices Test (Raven, 2003) was employed to assess fluid intelligence via a series of multiple‐choice questions that adhere to consistent principles and utilize perceptual analogies in matrix form, with each row and column connected by effective relationships. The lower right‐hand corner of each matrix is absent. The child must select the missing component from six possible images to complete a geometric figure; the complexity of patterns increases progressively across 36 items presented.

##### Socio‐economic status

The impact of physical activity on children's development may be influenced by their SES (Raudsepp, 2006). Research has demonstrated that SES is associated with EF skills (Ming et al., 2021) and academic achievement (Broer et al., 2019). Hence, it is imperative to evaluate whether the SES of the two cohorts of children were equivalent at baseline. Socioeconomic data were obtained via a sociolect‐demographic questionnaire, which was completed by parents, who provided written informed consent. A child's SES was determined based on the parents' educational attainment (rated as 1 for primary school level, 2 for junior high school level, 3 for high school level, and 4 for university level) and occupational classification (rated as 1 for unemployed or unskilled work, 2 for blue‐collar or service industry, 3 for a medium‐level professional role, and 4 for a senior professional role). The SES score is calculated as the sum of the highest academic and professional scores of either parent, with a maximum total score of 8.

##### Parent report questionnaire

The attention deficit hyperactivity disorder (ADHD) Diagnostic Scale‐Parent Version (Su et al., 2006) was utilized as a baseline assessment tool to determine the presence of differences in inattention and hyperactivity behaviors between the intervention and control groups. Parents utilized a Likert scale to assess the frequency of their children's symptoms related to inattention, behavioral problems, and hyperactivity. The scale ranges from 0 (*never*) to 3 (*very frequent*).

##### Body mass index

The children's weight (kg) and height (cm) were measured, and BMI was included as a covariate because of evidence linking it to cognitive impairment (Hillman et al., 2015).

#### Statistical analysis

The experimental data were analyzed using SPSS 21.0 software (IBM, Armonk, New York, USA). First, a descriptive analysis was performed on all data to assess the normality of variable distribution and identify missing values and significant outliers. Subsequently, the demographic and behavioral characteristics between the two groups were compared using the *χ* ^2^ test and *t* test. Additionally, the score of the Raven test, SES, BMI, and pretest score was utilized as a covariate to examine the impact of intervention on EF in children, with Cohen's *d* being employed to determine effect size. The magnitude of Cohen's *d* falls within the range of small‐effect (0 to 0.2), moderate‐effect (0.2 to 0.8), and large‐effect (exceeding 0.8) categories, as defined by Cohen (2013).

## RESULTS

During the entire intervention period, there were no reported injuries, and the adherence rate was 94.6%. Descriptive statistics revealed no floor or ceiling effects in EF task performance, with missing values ranging from 0% to 4%. Scores that deviated from the average by three or more standard deviations, which are considered outliers, were excluded from the analysis. The percentage of outliers across all tasks fell within the range of 0% to 1%. All data conform to a normal distribution.

According to the data presented in Table 2, there were no significant differences in EF task performance between the two groups at baseline. With regard to EF task performance, except for the stop signal task, there was no statistically significant difference observed between the intervention and control groups. However, in terms of stop signal task accuracy, the control group exhibited superior performance compared with the intervention group. To further assess the efficacy of our intervention, we utilized pretest scores for each EF task as a covariate and conducted covariance analysis to compare EF performance between the two groups.

**TABLE 2:** Comparison of EF task performance between the IG and CG at baseline level, M (*SD*).

Variables	IG	CG	Difference	
Delay gratification				
Snack delay	7.44 (1.25)	7.82 (1.22)	*t*_(1,61)_ = −0.825, *p* = .279	ND
Wrapped gift time	28.69 (19.40)	28.33 (20.14)	*t*_(1,61)_ = 0.433, *p* = .535	ND
Wrapped gift strategy	2.74 (1.23)	2.52 (1.27)	*t*_(1,61)_ = 0.760, *p* = .627	ND
Inhibitory control				
Stop signal time	861.45 (107.43)	842.37 (105.68)	*t*_(1,63)_ = 0.416, *p* = .643	ND
Stop signal accuracy	50.43 (11.02)	57.06 (13.54)	*t*_(1,63)_ = −1.732, *p* = .041	CG > IG
Circle drawing	0.54 (0.22)	0.42 (0.25)	*t*_(1,61)_ = 1.331, *p* = .283	ND
Working memory				
Letter memory	2.23 (0.32)	2.45 (0.37)	*t*_(1,63)_ = −0.436, *p* = .681	ND
Keep track	2.91 (1.73)	3.87 (1.81)	*t*_(1,63)_ = −2.017, *p* = .157	ND
Cognitive flexibility				
Go/No‐Go time	803.65 (178.82)	773.15 (175.59)	*t*_(1,63)_ = 0.764, *p* = .347	ND
Go/No‐Go accuracy	27.59 (6.62)	31.03 (6.93)	*t*_(1,63)_ = 1.266, *p* = .842	ND
Dots time	1284.22 (287.62)	1122.38 (291.26)	*t*_(1,63)_ = 0.732, *p* = .837	ND
Dots accuracy	68.05 (12.75)	65.15 (12.40)	*t*_(1,63)_ = 0.245, *p* = .729	ND

Abbreviations: CG = control group; IG = intervention group; M (*SD*) = mean (standard deviation); ND = no difference.

The results of the covariance analysis indicate a significant difference in the experimental outcomes, with baseline data for the following tasks being controlled (Table 3). The Snack delay exhibited a significant effect (*F*_(1,60)_ = 7.43, *p* = .002, *d* = .66), as did the Wrapped gift time (*F*_(1,59)_ = 7.85, *p* = .001, *d* = .43), Stop signal time (*F*_(1,61)_ = 3.85, *p* = .026, *d* = .55), Circle drawing task (*F*_(1,61)_ = 7.24, *p* = .002, *d* = .54), Letter memory task (*F*_(1,61)_ = 6.20, *p* = .031, *d* = .49), Keep track task (*F*_(l,61)_ = 6.43, *p* = .001, *d* = .62), Dots time (*F*_(l,6L)_ = 3.65, *p* = .027, *d* = .59), and Dots accuracy (*F*_(l,6L)_ = 7.44, *p* = .031, *d* = .70).

**TABLE 3:** Comparison of EF task performance between the IG and CG at post‐assessment, M (*SD*).

Variables	Post‐assessment		Group effect			
	IG	CG	*F*	*p*	Cohen's *d*	Comparison
Snack delay	10.41 (1.62)	9.32 (1.64)	7.43**	.002	.66	IG > CG
Wrapped gift time	26.13 (20.07)	34.78 (20.40)	7.85**	.001	.43	CG > IG
Wrapped gift strategy	2.88 (1.24)	2.65 (1.26)	2.51	.171	.18	ND
Stop signal time	873.18 (113.25)	813.22 (102.64)	3.85*	.026	.55	IG > CG
Stop signal accuracy	67.25 (12.55)	71.68 (10.25)	0.49	.065	.39	ND
Circle drawing	0.62 (0.27)	0.47 (0.29)	7.24**	.002	.54	IG > CG
Letter memory	2.43 (0.34)	2.27 (0.31)	6.20*	.031	.49	IG > CG
Keep track	4.92 (1.87)	3.81 (1.62)	6.43**	.001	.62	IG > CG
Go/No‐Go time	766.34 (185.42)	758.26 (173.55)	0.24	.143	.04	ND
Go/No‐Go accuracy	42.81 (6.48)	43.97 (6.66)	0.54	.351	.18	ND
Dots time	1265.32 (282.27)	1084.22 (328.45)	3.65*	.027	.59	IG > CG
Dots accuracy	75.70 (10.33)	68.10 (11.27)	7.44*	.031	.70	IG > CG

*Note*: Asterisks represent significant differences.

Abbreviations: CG = control group; IG = intervention group; M (*SD*) = mean (standard deviation); ND = no difference.

* *p* < .05;

** *p* < .01.

The children who engaged in recreational gymnastics demonstrated superior performance compared with the control group across most tasks assessing inhibition control, working memory, and cognitive flexibility. However, mixed results were observed in hot EF tasks. Specifically, in the Snack delay task, children receiving the intervention demonstrated longer delay times than their counterparts in the control group. In the Wrapped gift task, the control group exhibited a significant increase in delayed gratification after the intervention, whereas no such effect was observed in the intervention group.

## DISCUSSION

Numerous cross‐sectional studies have explored the correlation between physical activity and cognitive function in both children and adults (Cox et al., 2021; Kouklari et al., 2018; Trudeau & Shephard, 2008). However, there is a paucity of research on the utilization of structured sports activities for enhancing the cognitive development of preschool‐aged children. This study represents one such intervention aimed at exploring the impact of recreational gymnastics on EF in Chinese preschoolers. The results of the covariance analysis indicate that the recreational gymnastics intervention program exerted a positive impact on the development of core components of EF, namely inhibition control, working memory, and cognitive flexibility. The intervention group exhibited superior inhibitory control, particularly in regulating continuous movement during the Circle task. They demonstrated shorter response times and higher accuracy under interference stimuli during the Stop signal task, while also performing better in working memory during both the Letter memory and Tracking tasks. Additionally, they displayed increased cognitive flexibility when switching between tasks during the Dots task.

In the hot EF tasks, the outcomes of the intervention did not align with initial expectations. Specifically, in the Snack delay task, children who received intervention exhibited longer delays compared with their control group counterparts. In contrast, during the post‐test phase of the Wrapped gift task, children in the control group demonstrated an increase in delayed gratification time, while those who underwent intervention did not exhibit such changes. This discrepancy may potentially indicate the impact of recreational gymnastics on diverse EF tasks and the variations in cognitive regulation associated with these tasks. This outcome may be influenced by a multitude of factors, some of which could stem from the intervention program itself or the intricacies of the study design, while others might be associated with the characteristics of the subject under intervention. First, the factors of the intervention program may influence the interpretation of the findings. Medium‐intensity recreational gymnastics can have a multifaceted and diverse impact on children's cognitive function. While recreational gymnastics itself contributes to enhancing physical and mental health in children, the specific types, duration, and frequency of gymnastics employed in this study may yield varying effects on EF. Furthermore, the personal motivation (Taylor et al., 2004), level of physical fitness (Nieto‐López et al., 2020), and experience of the children's participation (Rosenberg, 2015) may also exert an influence on both the magnitude and direction of the intervention effect. Second, factors related to research design and implementation may also affect the interpretation of the research results. Although the randomized controlled experiment used in this study is a robust research design, other potential confounding variables such as sample size and representativeness and intervention quality may cause the results to deviate from the hypothesis. In addition, the effectiveness and sensitivity of the measurement tools may also be an important factor in this unexpected result. Third, individual differences and environmental factors may be important factors that cannot be ignored in this unexpected result. Children's age (Huizinga et al., 2006), sex (Grissom & Reyes, 2019), family background, and SES (Ursache & Noble, 2016) could potentially impact the magnitude and direction of the intervention effect in recreational gymnastics. In addition, other factors in the research environment, such as the school climate (Piccolo et al., 2019), family support (Schroeder & Kelley, 2010), and community resources, may also be important factors contributing to such unexpected results. In conclusion, the inconsistency observed between the research findings and the hypothesis in the hot EF tasks may be attributed to an interplay of multiple factors. In the future, we will conduct further investigations to explore the intricate relationship between these factors, aiming to acquire a comprehensive understanding of the underlying causes behind these unexpected outcomes and provide valuable insights for future intervention programs and research design.

In this study, the group of children who received recreational gymnastics intervention demonstrated superior performance on most tasks related to EF compared with the control group. The observed discrepancy between the intervention and task‐specific training effects suggests that preschoolers who participated in the program experienced genuine enhancements in their executive functioning abilities. Below, we will explore potential reasons why recreational gymnastics interventions may improve EF among young children.

First, the effectiveness of the intervention program may be attributed to the cognitively enriched physical activity, which not only induces physiological arousal in children but also activates cognition‐related neural networks to a greater extent. When children are faced with difficult, unfamiliar, and constantly changing motor skills or situations, it can facilitate pre‐activation of the neural network in the cerebellar and prefrontal regions (Colcombe et al., 2004), thereby enhancing their performance on EF tasks. Previous intervention studies have demonstrated that physical activity rich in cognitive stimuli is more effective for improving EFs than physical activity without such stimuli (Pesce et al., 2016; Schmidt et al., 2015). In the intervention plan of this study, we augmented the cognitive load of the intervention task, requiring participants to engage in specific cognitive processes to attain task objectives. For instance, in the game of traversing a log bridge, two players traverse the balance beam from opposite ends and constantly adjust their bodies to maintain equilibrium before meeting at the center; upon convergence, they must collaborate and strategize to overcome obstacles en route to reaching the finish line. This sequence of motor processes necessitates continual bodily adjustment and control, activating the potential neural network of the prefrontal cortex and other associated regions. Consequently, participation in recreational gymnastics has been shown to enhance children's EF. The findings of this investigation corroborate a recent study that implemented systematic training involving high‐level cognitive processes, resulting in significant enhancements in inhibitory control, working memory, and cognitive flexibility among young children (Bayanova et al., 2022).

Engaging in complex motor coordination activities may enhance children's EF performance by facilitating cognitive involvement in physical pursuits (Best, 2010). Coordinated physical activities encompass a diverse range of movements and skills, ranging from fundamental actions such as walking, running, and jumping to more intricate ones such as recreational gymnastics (Werner et al., 2012). These activities necessitate proficient neuromuscular coordination to ensure precise timing and intensity of bodily functions in order to accomplish specific movements or tasks. Coordination also entails maintaining balance and spatial awareness to ensure stability during exercise and adaptability to varying environmental conditions (Horak, 2006). The adaptability of coordinated exercise enhances the neuromotor ability of both the peripheral nervous system (e.g., neuromuscular ability) (Mulder & Hochstenbach, 2001) and the central nervous system (e.g., brain neural circuitry) (Shepherd, 2001), thereby providing a constructive foundation for cognitive improvement. The recreational gymnastics movements employed in this study were carefully selected by experienced gymnastics instructors based on the physical and cognitive developmental characteristics of preschool children. For example, they included different forms of walking on a balance beam (height 50 cm), different forms of jumping on a small trampoline (height 30 cm), etc. These movements are coordinated physical activities. They may also enhance cognitive engagement among children during the intervention, thereby promoting their development of EFs.

Second, another important factor contributing to the improvement of EF is moderate‐intensity physical activity. Meta‐analysis has shown that chronic moderate‐intensity physical activity can have the greatest positive impact on human cognitive function (Etnier et al., 2006; Knaepen et al., 2010). A significant amount of evidence suggests that the effect of exercise training on cognition is mediated by neurotrophic factors (Cotman & Berchtold, 2002). However, the transcription of these neurotrophins was found to be attenuated during excessive physical activity (Lou et al., 2008; Schaaf et al., 2000), indicating that strenuous exercise may not necessarily improve cognitive function and could potentially cause biochemical damage. Furthermore, evidence suggests that efforts to increase moderate to vigorous physical activity in early childhood may compete with energy expenditures associated with brain development, which could interfere with the development of EF skills (Voss et al., 2014).

The potential mechanisms underlying the improvement of preschoolers' EF through recreational gymnastics may involve physiological changes, such as alterations in the nervous system, and social interactions, including the social experiences facilitated by participation in recreational gymnastics. Recreational gymnastics has the potential to impact preschool children's EF through promoting neuroplasticity and neuroregulation, resulting in positive physiological changes within their nervous systems (Hart & Zernicke, 2020). First, engaging in recreational gymnastics requires preschoolers to perform a variety of coordinated movements, potentially leading to the formation and strengthening of new connections between neurons (Myer et al., 2015), a phenomenon known as neural plasticity. This plasticity renders children's neural networks more flexible and efficient at adapting to learning and executing novel tasks. Second, participating in gymnastic exercises can have a beneficial influence on children's neuroregulation (Caine et al., 2013). Precise muscle control and coordinated movements are essential during gymnastic routines, thereby enhancing efficiency within the neural system for performing and adjusting movements (Iorga, 2019). Through repeated practice of these movements, the nervous system gradually establishes a more refined and coordinated neural control model – an aspect crucial for improving EFs such as motion control, coordination, and reaction time. In general, recreational gymnastics can elicit neurophysiological responses in children, facilitating the occurrence of neural plasticity and enhancing neural regulation, thereby providing valuable support for the EF of preschoolers. This physiological adaptation may represent one of the pivotal mechanisms through which recreational gymnastics exerts a positive impact on the holistic development of preschool children.

Another potential mechanism through which recreational gymnastics positively impacts the EF of preschool children is by enhancing their capacity for emotional regulation. Recreational gymnastics incorporates a range of sensory movements and movement control in its athletic requirements (Garcia et al., 2011), making it not only a type of physical training but also an emotional and cognitive challenge. First, recreational gymnastics provides an outlet for emotional expression and release (McCaw, 2012). Through exercise, children can express and release their emotions within a safe and engaging environment, which aids the regulation of their emotions. Physical activity in sports helps alleviate tension and promotes both physical and emotional equilibrium (Hansmann et al., 2007). Second, gymnastic exercises foster children's ability to cope with challenges (Burgess et al., 2016). Accomplishing intricate gymnastic movements necessitates a constant overcoming of difficulties, often accompanied by setbacks as well as successes. By gradually surpassing their limits, children acquire patience and persistence when faced with obstacles, thus cultivating positive strategies for emotion regulation. In general terms, recreational gymnastics offers a multi‐faceted and comprehensive experience that encompasses physical, emotional, and cognitive aspects. Through avenues such as emotional expression, conquering challenges, and social cooperation, children develop robust skills for emotion regulation within the realm of recreational gymnastics, potentially serving as a significant medium for enhancing their EF.

## LIMITATIONS AND FUTURE DIRECTIONS

Although our research has yielded significant results, there are still certain limitations. First, despite the implementation of a randomized controlled trial design, potential heterogeneity between groups may impact the experimental outcomes. For instance, owing to sample‐size constraints, variations in sex, age, or health status could influence the study findings. Moreover, potential measurement deviations during the experiment might affect internal validity. Despite employing standardized measurement tools and rigorous training to standardize the process, confounding factors such as individual differences, measurement errors, or evaluator biases may still have existed and influenced the study findings. Second, we assessed only the core components of EF in young children, without conducting a comprehensive evaluation of other aspects within this domain. Additionally, we did not thoroughly investigate the impact of the intervention quality on outcomes. For example, disparities in children's participation rates and adherence to intervention programs may affect their effectiveness. In addition, we have not explored the potential mechanisms for implementing functional improvements. Finally, the generalizability of our research findings is limited. Given that our research samples consisted primarily of 5–6‐year‐old kindergarten children from specific regions, the applicability of our results to children across other regions, cultural backgrounds, and age groups might be restricted.

In the future, we will employ a larger sample size and utilize diverse methodologies to further validate the impact of moderate‐intensity gymnastics on the EF of young children, as well as investigating its potential mechanisms and generalizability. Moreover, we will incorporate additional control variables into our research design to mitigate potential confounding factors and adopt more comprehensive measurement tools to enhance the reliability of our findings.

## CONCLUSION

The findings of this investigation suggest that engaging in moderate‐intensity recreational gymnastics may facilitate the development of EF among preschool‐aged children. Future research endeavors featuring larger, more representative sample sizes and longer durations are better suited to ascertain the dose–response relationship and underlying mechanisms by which recreational gymnastics promotes EF development in young children.

## CONFLICT OF INTEREST STATEMENT

The authors declare there are no conflicts of interest.

## ETHICS STATEMENT

The present study has obtained approval from the Ethics Committee for Exercise Science Experiments at Beijing Sport University (Agreement code 2022015H). Prior to commencing the study, all participating children and their guardians were provided with comprehensive information regarding the study's objectives, procedures, potential risks and benefits, and subsequently provided written informed consent. Throughout the entire duration of the study, strict adherence to ethical principles was maintained in order to safeguard participant privacy and personal information; furthermore, all data were processed anonymously. This investigation adheres to the ethical guidelines outlined in the Helsinki Declaration to ensure participant safety and well‐being.

## Data Availability

Relevant data can be furnished in accordance with the stipulated requirements.
